# Correlation of anterior segment optical coherence tomography measurements with graft trephine diameter following descemet stripping automated endothelial keratoplasty

**DOI:** 10.1186/1471-2342-12-19

**Published:** 2012-07-23

**Authors:** Gavin S Tan, Mingguang He, Donald T Tan, Jodhbir S Mehta

**Affiliations:** 1 Singapore National Eye Centre (SNEC), 11 Third Hospital Avenue, Singapore, 168751, Singapore; 2 Singapore Eye Research Institute (SERI), 11 Third Hospital Ave, Singapore, 168751, Singapore; 3 Zhongshan Ophthalmic Centre, Guangzhou, China; 4 Yong Loo Lin School of Medicine, National University of Singapore, 1E Kent Ridge Road, Singapore, Singapore; 5 Department of Clinical Sciences, Duke-NUS Graduate Medical School, 8 College Rd, Singapore, 169857, Singapore

## Abstract

**Background:**

To assess repeatability of the Zhongshan Assessment Program (ZAP) software measurement of Anterior Segment Optical Coherence Tomography (ASOCT) images and correlate with graft trephine diameter following Descemet Stripping Automated Endothelial Keratoplasty (DSAEK)

**Methods:**

Retrospectively evaluated interventional case series. 121 consecutive eyes undergoing DSAEK over a 26 month period underwent ASOCT imaging 1month after their surgery. ASOCT images were processed using ZAP software which measured the graft and cornea parameters including anterior and posterior graft arc length and cord length, posterior cornea arc length (PCAL) and anterior chamber width.

**Results:**

The graft measurements showed good repeatability on ASOCT using ZAP with high intra class coefficient and small variation in the coefficient of variation. On ASOCT, the mean recipient PCAL was 12.99+/−0.69mm and the anterior chamber width was 11.16+/−0.57mm. The mean Graft anterior arc length was 9.69+/−0.66mm and the mean Graft anterior cord length was 8.92+/−2.94mm. The mean graft posterior arc length was 9.24+/−0.75mm and the mean graft posterior cord length was 8.15+/−0.57mm. Graft posterior arc length (rho=0.788, p< 0.001) correlated best with intra-operative graft trephine diameter. The mean ratio of posterior graft arc length to PCAL was 0.712 +/− 0.056.

**Conclusions:**

We have validated the repeatability of the ZAP software for DSAEK graft measurements from ASOCT images and shown that the graft arc length parameters calculated from the ASOCT images correlate well with the intra-operative graft trephine diameter. This software may help surgeons determine the optimal DSAEK graft size based on pre-operative ASOCT measurements of the recipient eye.

## Background

Descemet stripping automated endothelial keratoplasty (DSAEK), the main form of endothelial keratoplasty in which a posterior lamellar graft is attached to the posterior corneal surface is rapidly becoming the surgical alternative to penetrating keratoplasty (PK) for patients with corneal endothelial failure [1,2]. The size of the graft diameter in DSAEK usually exceeds that of conventional PK, and a 9.0mm graft, which is often possible in DSAEK, [1] will transfer 26% more surface area of healthy donor endothelial cells than a standard 8.0mm graft more commonly used in PK [3-5]. Although larger grafts offer the inherent advantage of transplanting more healthy donor endothelial cells, a larger diameter graft in DSAEK is technically more difficult to insert with an increased the risk of surgical graft trauma, and may crowd the chamber angle resulting in peripheral anterior synechiae (PAS) in eyes with shallow anterior chambers. Graft diameter has also been reported to have a small but statistically significant correlation with hyperopic shift post operatively [6,7].

Anterior segment optical coherence tomography (ASOCT) is a non-contact imaging technique that obtains high-resolution cross-sectional images of the cornea and anterior chamber. The ability of anterior segment OCT to render tissue planes with high axial resolution is potentially useful in evaluating the cornea after corneal lamellar procedures [8]. ASOCT has been used after DSAEK for assessing corneal deturgescence [9], predicting primary graft failure [10] and for quantitative imaging of the donor lamella and its anatomic effects within the anterior chamber [8].

The aim of this study was to (i) assess the reproducibility of post DSAEK graft measurements using the modified ZAP (Zhongshan Assessment Program) software (ii) to validate the measurements obtained from the ZAP readings and correlate this with intra-operative graft trephine measurements; (iii) to retrospectively analyze the graft sizing in our first 121 DSAEK cases to assess the size of the DSAEK graft in relation to the posterior corneal arc length (PCAL).

## Methods

Approval for the study was granted by the Singapore National Eye Centre and Singapore Eye Research Institute institutional review board. The study was conducted in accordance with the Declaration of Helsinki. Written informed consent was obtained from all subjects before enrollment. One hundred and twenty one consecutive eyes undergoing DSAEK at the Singapore National Eye centre over a 26 month period, performed by a single corneal surgeon (DT), had ASOCT imaging performed one month after their surgery.

### DSAEK surgical technique

DSAEK was performed using a similar technique, as described by Price [11], with the exception of the method of donor insertion. The taco folding technique was used in the first 20 eyes [12], followed by our previously described Sheets Glide technique in all subsequent 101 cases [13]. Microkeratome dissection of the donor was performed using an automated lamellar therapeutic keratoplasty unit (Carrazio-Barraquermicrokeratome, Moria). The diameter of the donor buttons ranged from 7.0 to 9.5mm with an increment of 0.25mm, and the trephine diameter was estimated at approximately 2mm less than the white-to-white measurement of the recipient corneal diameter, as measured by standard surgical calipers intra operatively. Graft thickness was determined by ultrasound pachymetry, after microkeratome dissection, prior to trephination.

### Anterior segment optical coherence tomography

Anterior Segment images were scanned with the Zeiss Visante ASOCT (Carl Zeiss Meditec, Dublin, CA) within the first month post operation, with informed consent obtained from all participants, as part of our IRB approved study on DSAEK. The details of ASOCT imaging technology have been described previously [14-16]. To correct for errors caused by the different refractive indices of the cornea and aqueous, the software integral to the Visante ASOCT, initially de-warps the image at the air–tear interface and at the corneal–aqueous interface. Images were taken directly from the machine’s output function as 816 x 636 pixel JPEG (lossless compression) files. All selected images were temporal/nasal i.e. horizontal scans, to maximize visibility of anatomical location and repeatability [17,18].

### Image processing

**Figure 1:**
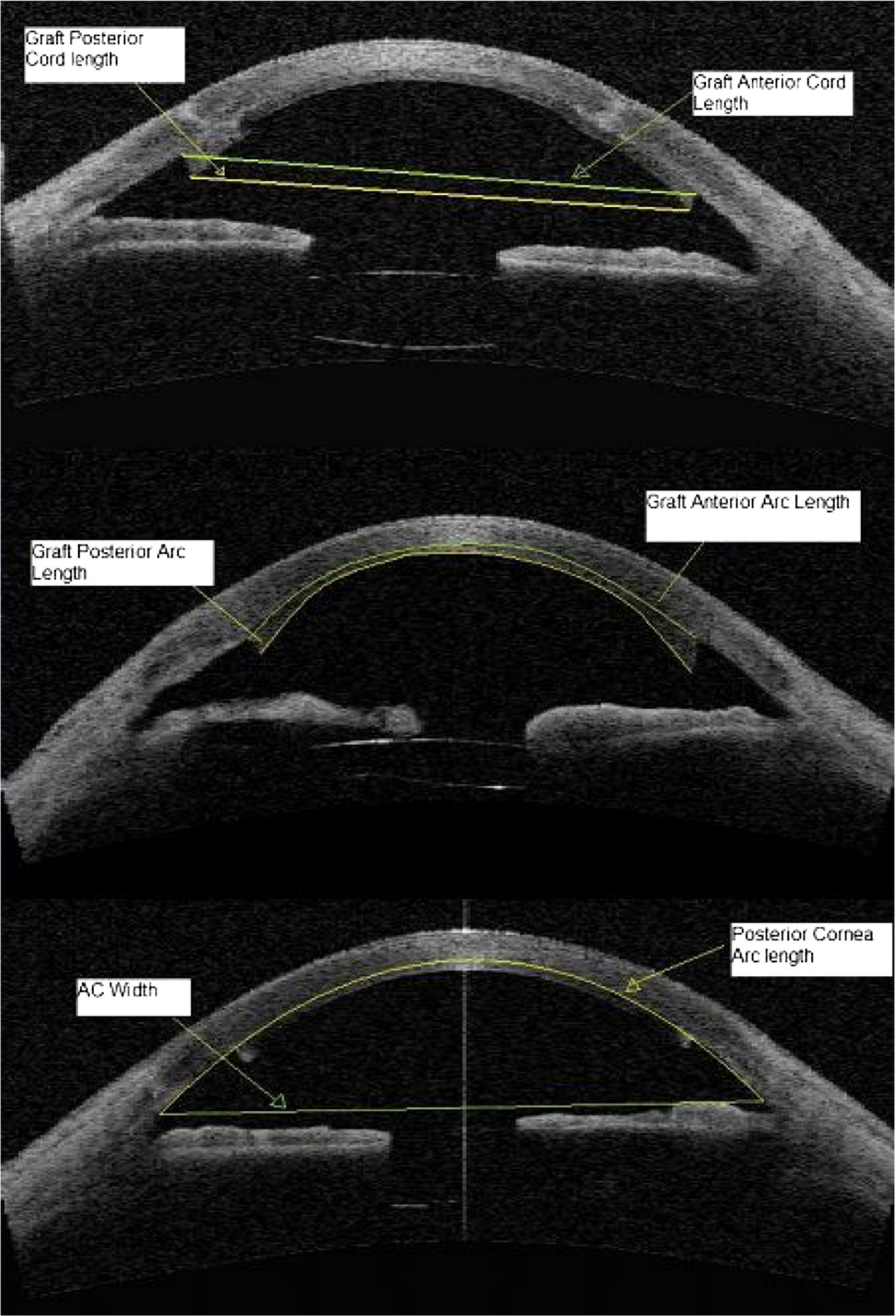
ASOCT image with markings and measurements.

All ASOCT images were assessed by one ophthalmologist (GT). For each image, the image file was opened using the Zhongshan Assessment Program and first, the two scleral spurs were identified, defined as the anatomical junction between the inner wall of the trabecular meshwork and the sclera (Figure 1) [19-21]. It is marked by a prominent inner extension of the sclera at its thickest part, and in this study it was defined as a change in curvature of the inner surface of the angle wall, often appearing as an inward protrusion of the sclera. Following this, the anterior and posterior lateral most edges of the DSAEK graft were marked, the software then calculated all parameters and the information was recorded (Figure 1).

The Zhongshan Assessment Program (ZAP, Guangzhou, China) is a proprietary non-commercially available anterior segment image processing software developed at the Zhongshan Ophthalmic centre, Guangzhou, China. This software may be requested free from the authors. The software automatically extracted the 300 x 600 8-bit greyscale (intensities from 0 to 255) image portion of the output file and performed noise and contrast conditioning [17]. A binary copy of the image was then produced where pixels were either 1 s (tissue) or 0 s (open space) depending on whether they were brighter or darker than a calculated threshold value. Algorithms defined the borders of the corneal epithelium and endothelium, and the anterior surface of the iris [17,22]. The anterior graft cord length and the posterior graft cord length were measured, defined as the straight line distance between the anterior edges and the posterior edges of the graft respectively. The anterior and posterior graft arc length were measured which represented the linear measurement of arc length of the anterior and posterior surfaces of the cornea. The posterior cornea arc length from scleral spur to sclera spur and the anterior chamber width using the scleral spurs as the landmarks were also measured. (Figure 1) Repeat measurements with the ZAP software were done for 20 randomly selected ASOCT images one month after the initial measurements were completed, with the observer masked to the results of the initial analysis to assess the intra-observer repeatability of the measurements.

### Statistical methods

Parametric and non-parametric tests were used to compare continuous variables according to data distribution. Spearman’s Rho correlation, linear regression and logistic regression analyses were used to assess factors relating to the posterior corneal arc length. P< 0.05 was considered statistically significant. Bland-Altman analysis was performed to analyze intra-observer agreement. Analysis of repeat measurements was done looking at limits of agreement, coefficient of variation and intraclass coefficient. Statistical analysis was performed by SPSS and microsoft® Excel software.

## Results

### Patient characteristics

**Figure 2:**
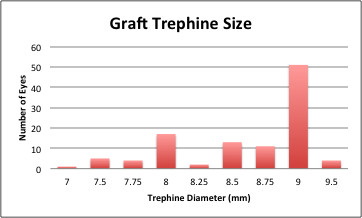
Distribution of Graft Trephine size.

A total of 121 eyes underwent DSAEK and had ASOCT done. 12 (9.9%) eyes were excluded because the ASOCT image was of insufficient quality to identify the sclera spur or to process the image with ZAP software. The remaining 109 eyes (56 right and 53 left) of 104 patients were included in this study. The mean age was 66.17+/−11.58 years and 53.2% were male (58 male patients & 46 female patients). The Sheets Glide insertion technique was used in 93 patients (85.3%) with acceptable ASOCT images. Mean pre-operative donor endothelial cell count was 2881.54 +/− 240.78 and the mean graft thickness was 194.50 +/− 43.10 um (Table 1). The graft trephine size used ranged from 7.0 to 9.5mm with a median and mode of 9.0mm (Figure 2).

**Table 1:** Demographics and graft parameters

	**N=109 (Mean +/− SD)**
Age (Years)	66.17 +/−11.58
Sex (Male)	58 (53.2%)
Side (OD)	56 (51.4%)
Glide Insertion technique	93 (85.3%)
Graft size (mm)	8.63 +/− 0.53
Endothelial Cell Count	2881.54 +/− 240.78
Graft thickness (um)	194.50 +/− 43.10
Posterior Cornea Arc length (mm)	12.99 +/− 0.69
Cornea Posterior Curvature (mm)	5.80 +/− 0.85
Posterior graft arc length (mm)	9.24 +/− 0.72
Posterior graft cord length (mm)	8.15 +/− 0.57
Anterior graft arc length (mm)	9.69 +/− 0.66
Anterior graft cord length (mm)	8.65 +/− 0.48
Ratio of posterior graft arc length to posterior Cornea arc length	0.712 +/− 0.516
Ratio of Anterior graft arc length to posterior Cornea arc length	0.745 +/−0.051
Ratio of posterior graft arc length to Posterior graft cord length	1.134 +/− 0.036
Ratio of anterior graft arc length to anterior graft cord length	1.122+/−0.048
Ratio of anterior graft arc length to posterior graft arc length	1.048 +/−0.048
Ratio of anterior graft Cord length to posterior graft cord length	1.096+/−0.339

### ASOCT analysis

#### Intra-observer repeatability of graft parameters

**Figure 3:**
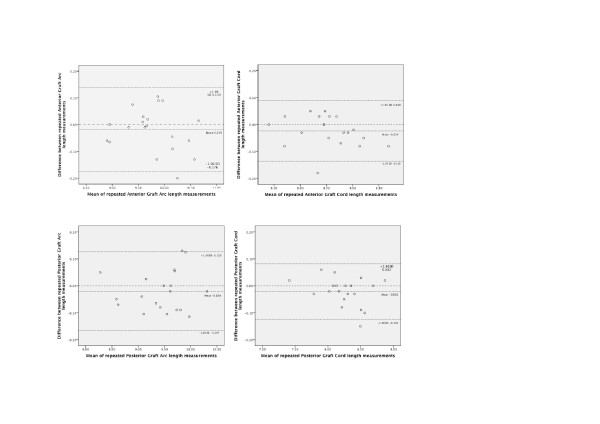
Band Altman Plots for Intraobserver agreement of Graft Parameters (Anterior graft Arc Length, Anterior Graft cord length, Posterior Graft Arc length, Posterior Graft Cord Length)

We have previously shown good repeatability for the measurement of recipient posterior cornea arc length[22] but not graft measurements. On repeated measurements of the graft parameters on ASOCT, there was no significant difference in the intra-observer measurements. There was high intra class coefficient amongst all parameters, corneal recipient arc length, anterior chamber width, anterior graft arc length, anterior graft cord length, posterior graft arc length and posterior graft cord length ranging from (0.900 to 0.989), which suggests a high level of agreement between repeated measurements. The coefficients of variation amongst all the graft parameters were small (1.24 to 3.71%) (Table 2). The Band Altman plots demonstrate that the mean difference between repeated measures are small and the limits of agreement are within an acceptable range (Figure 3) .

**Table 2:** Repeatability of ASOCT graft measurements

**Parameter**	**Mean (SD)**	**Mean (SD)**	P*	**Mean difference (95%CI)**	**Limits of Agreement**	**Coefficient of variation (95% CI)**	**Intraclass Coefficient (95% CI)+**
	**Observer 1**	**Observer 2**					
Posterior Cornea Arc length (mm)	13.08 (±0.61)	13.06 (±0.59)	0.634	0.02 (−0.06 to 0.10)	−0.32; 0.35	2.56% (1.50, 3.63)	0.959 (0.899 to 0.983)
Anterior chamber Width (mm)	11.24 (±0.50)	11.24 (±0.45)	0.942	0.00 (−0.10 to 0.10)	−0.42; 0.41	3.71% (0.25, 5.25)	0.900 (0.765 to 0.959)
Ant Graft Arc length (mm)	9.77 (±0.51)	9.783 (±0.53)	0.317	−0.02 (−0.06 to 0.02)	−0.18; 0.14	1.61% (0.94, 2.28)	0.988 (0.970 to 0.995)
Ant Graft Cord length (mm)	8.70 (±0.33)	8.73 (±0.34)	0.075	−0.02 (−0.05 to 0.00)	−0.14; 0.09	1.28% (0.75, 1.81)	0.985 (0.963 to 0.994)
Post Graft Arc length (mm)	9.43 (±0.52)	9.45 (±0.51)	0.240	−0.02 (−0.06 to 0.02)	−0.17; 0.13	1.55% (0.91, 2.19)	0.989 (0.973 to 0.996)
Post Graft Cord length (mm)	8.24 (±0.32)	8.26 (±0.34)	0.088	−0.02 (−0.05 to 0.00)	−0.12; 0.08	1.24% (0.73, 1.76)	0.988 (0.969 to 0.995)

*(2-sided).

+ all p< 0.001.

### Endothelial keratoplasty analysis

On ASOCT, the mean recipient posterior cornea arc length was 12.99 +/− 0.69mm and the mean anterior chamber width was 11.16 +/− 0.57mm. The mean Graft anterior arc length was 9.69 +/− 0.66mm and the mean Graft anterior cord length was 8.92 +/− 2.94mm. The mean graft posterior arc length was 9.24 +/− 0.75mm and the mean graft posterior cord length was 8.15 +/− 0.57mm. The mean ratio of posterior graft arc length to posterior cornea arc length was 0.712 +/− 0.056 (range 0.514 to 0.849) and the mean ratio of anterior graft arc length to posterior cornea arc length was 0.745 +/−0.051 (range 0.618 to 0.859).

**Figure 4:**
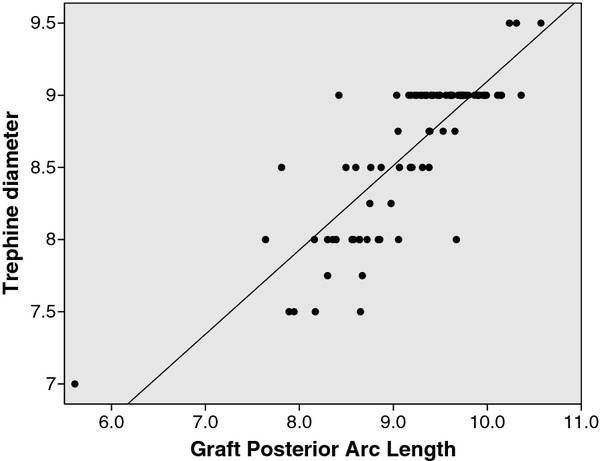
Correlation between trephine diameter and graft posterior arc length.

Bivariate correlation showed that intraoperative graft trephine diameter correlated with graft anterior arc length, graft anterior cord length, graft posterior arc length and graft posterior cord length (all p< 0.001) (Table 3). The intraoperative graft trephine diameter correlated best with graft posterior arc length (Figure 4). Multivariate linear regression analysis was performed with graft posterior arc length as the dependent parameter in order to analyse the relationship with trephine diameter (Table 4). Models were analyzed using all the parameters significant on bivariate correlation together with age and sex. After performing forward and backward selection multivariate models, we found the model including cornea posterior curvature, AC width and trephine diameter had the greatest adjusted R^2^ (0.697) for determining posterior graft arc length.

**Table 3:** Correlation between graft trephine diameter and ASOCT parameters measured

**ASOCT Parameter**	**Spearman’s Rho**	**p**
Posterior Cornea Arc Length	0.298	<0.001
Cornea posterior curvature	0.011	0.909
Anterior Chamber Width	0.162	0.094
Graft Anterior Arc length	0.681	<0.001
Graft Anterior Cord Length	0.643	<0.001
Graft Posterior Arc length	0.788	<0.001
Graft Posterior Cord Length	0.636	<0.001
Graft Thickness	−0.167	0.088

**Table 4:** Multivariate regression between Graft Arc Length and Trephine diameter

**Independent variables in regression model**	B coefficient* (95%CI)	**P value**	**Adjusted R**^**2**^
Recipent age, sex, posterior cornea arc length	1.112 (0.935, 1.289)	<0.001	0.677
Recipent age, sex, posterior cornea arc length, AC width	1.134 (0.958, 1.310)	<0.001	0.686
Recipent age, sex, posterior cornea arc length, AC width, thickness	1.134 (0.955, 1.312)	<0.001	0.683
Recipent age, sex, Cornea posterior curvature	1.162 (0.999, 1.324)	<0.001	0.682
Cornea Posterior curvature	1.154 (0.998, 1.310)	<0.001	0.685
AC width	1.117 (0.959, 1.276)	<0.001	0.691
Recipent age, sex, Cornea posterior curvature, posterior cornea arc length, AC width	1.122 (0.951, 1.292)	<0.001	0.694
Cornea Posterior curvature, AC width**	1.112 (0.954, 1.269)	<0.001	0.697

*B coefficient for trephine diameter.

**Final model using backward selection.

All variables in the models were tested for colinearity with acceptable tolerence.

**Figure 5:**
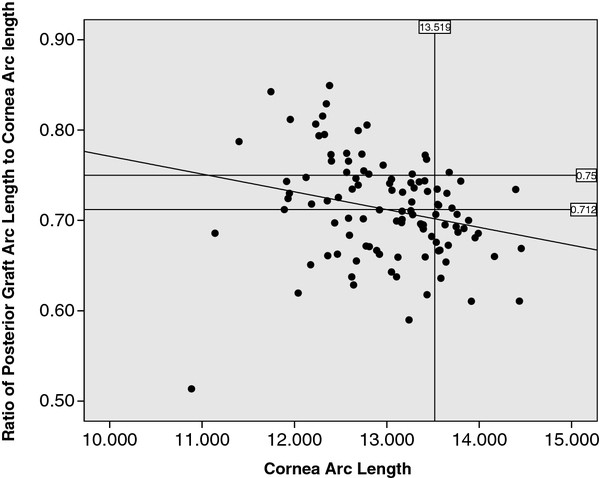
**Graph of Ratio of posterior graft arc length to posterior cornea arc length vs cornea arc length.** Vertical line represents the 75^th^ percentile of posterior cornea arc length (13.52mm). Horizontal line represents the mean ratio of graft posterior arc length to posterior cornea arc length (0.71).

Examining the trend of graft size, we found that the posterior graft arc length increased with posterior cornea arc length. However, we noted that in our series, eyes with larger posterior cornea arc length, the ratio of graft posterior arc length to posterior cornea arc length was less than eyes with smaller posterior cornea arc length. (Figure 5).

## Discussion

We have previously shown the utility of the ZAP software in providing reproducible measurements of posterior cornea arc lengths from ASOCT images [22]. In this study, ZAP was used to measure the graft parameters in ASOCT images of post-DSAEK eyes. We performed blinded intra-observer repeated measurements with the ZAP software on ASOCT images of endothelial keratoplasty in order to ascertain the repeatability of graft measurements which found that ZAP was able to provide reproducible measurements of DSAEK graft parameters from ASOCT images.

Although useful in providing anatomical detail of anterior segment structures, ASOCT does have its limitations. Images need to be dewarped with software algorithms to correct for index transitions [23], and calculations of anterior segment dimensions are also dependent on assumptions of cornea refractive index[24]. The time-domain ASOCT scans at 2000 axial lines per second and patient movement will affect the quality of the image and the accuracy of the dimensions measured from ASOCT scan. Therefore quantitative imaging on ASOCT may not always correlate with the actual tissue measurements. Our study compared an ex-vivo tissue measurement (the trephine diameter of the DSAEK graft), with the arc length of the graft measured post operatively on ASOCT. We found that among the in-vivo ASOCT graft measurements taken, the graft posterior arc length correlated best with graft trephine diameter. This may be expected since it would be less affected by the recipient cornea posterior curvature or the thickness of the graft, and the donor trephinations were all performed from the endothelial surface downwards.

Most of the grafts in this series were performed in eyes with pseudophakic bullous keratopathy where larger grafts with greater replacement of functional cornea endothelial cells were desirable. In our series, the mean ratio of posterior graft arc length to recipient posterior cornea arc length was 0.712 +/− 0.056 (Range 0.504 to 0.852). There was a small negative trend between the ratio of posterior graft arc length to posterior cornea arc length and the posterior cornea arc length, which suggested that we could have used larger grafts in eyes with larger posterior corneal arc lengths (Figure 5). For eyes in our series where the posterior cornea arc length was larger than the 75^th^ percentile (13.52mm), the ratio of graft posterior arc length to posterior cornea arc length was less than the mean (0.712) in 70.4% of these eyes. This suggests that 70.4% of grafts in patients with PCAL> 13.5mm were undersized.

We feel that sizing of the DSAEK graft is not a “one size fits all” procedure. It is known that cornea dimensions can vary with ethnicity and adult stature [25]. Intra-operative graft sizing based on visual assessment of the horizontal white to white diameter does not take into account the cornea curvature, and may also be an underestimate in eyes with significant arcus or pannus. The main advantage of using the ZAP software is that it allows the surgeon to quantitatively assess the size of the recipient posterior corneal surface as opposed to the anterior surface. Using the anterior surface of the cornea may be appropriate for sizing for penetrating keratoplasty but it is inappropriate for EK surgery not only due to the position of the EK graft but also because the EK surgeon can potentially transplant a larger graft since there is no concern regarding the limbal vasculature. There is no published information as to the optimal graft size for endothelial keratoplasty and hence also the optimal ratio of graft posterior arc length to posterior cornea arc length, although it has been suggested that a larger graft with the same endothelial cell density would provide a greater total number of functioning endothelial cells to the recipient and may support greater long term endothelial cell survival [26,27]. Although one retrospective study demonstrated no significant difference in the loss of endothelial cell density comparing 8mm grafts and grafts larger than 8mm [28], this study only included patients with Fuch’s endothelial dystrophy where the endothelial dysfunction is mainly in the central cornea and larger grafts may provide less of an advantage. Comparative studies between DSAEK and PK have shown that although DSAEK has a greater initial endothelial cell loss [29,30], the subsequent cell loss is at a slower rate in comparison to PK [26]. This may be related to the larger grafts with greater number of cells inserted during DSAEK. A large graft is not always an advantage, as an oversized graft may be more difficult to insert, and is also less tolerant to decentration, with a higher risk of crowding of the anterior chamber angle, contact with the peripheral iris leading to the development of peripheral anterior synechiae, and hence the potential for a higher risk of both angle-associated secondary glaucoma, and allograft rejection. The importance of DSAEK graft sizing may be of greater significance in our population of Asian eyes where the incidence of narrow angles and angle closure glaucoma is much higher than in Western and European populations [31]. Studies have also found that DSAEK grafts induce an initial hyperopic shift that decreases somewhat over time [32]. This hyperopic shift is reported to be correlated with central graft thickness, graft trephine diameter as well as the thickness gradient between the centre and periphery of the graft [6,7,33]. Examining the relationship between the posterior and anterior graft arc length, in addition to the graft thickness and diameter, with the final refractive outcome, will allow us to better predict this hyperopic shift with the ZAP software.

In future, we aim to perform further studies to prospectively analyze the effect of graft size and the ratio of graft diameter to posterior cornea arc length, on postoperative outcomes including endothelial cell count and refractive outcome. Once we establish an optimal graft size ratio, we can estimate the appropriate graft trephine diameter based on the cornea posterior arc length from the pre-operative ASOCT image. Our multiple regression analysis showed that, a model using posterior cornea arc length and AC width measured on ASOCT would allow us to reasonably estimate the graft arc length based on the trephine diameter chosen (Table 4).

The use of ASOCT imaging and ZAP software in our study does have some limitations. It has previously been shown that there can be difficulty in detecting the sclera spurs on some ASOCT scans [34]. In some images, the internal surface of the sclera formed a smooth continuous line with no inward protrusion or change in curvature which made it impossible to detect the sclera spur. In addition, some ASOCT images were of suboptimal quality, which also affects the ability to accurately identify the sclera spur. Some of these poor quality images could not be processed by the ZAP software even if the sclera spur could be identified; this figure was 9.9% in our study. The study was also limited in that measurements were performed on horizontal (nasal/temporal) ASOCT scans only. We chose to use only horizontal scans since these scans have been shown to be the most consistent with respect to obtaining high quality images for ZAP software analysis, and also recognizing that most surgeons measure the horizontal rather than vertical white-to-white diameter [19]. In addition, ASOCT imaging of vertical scans can be limited by eyelid anatomy especially in our population of Asian eyes with small palpebral apertures. A further limitation to the software is that it can only be used on time domain scans that image the whole length of the anterior segment. The next generation of Fourier domain ASOCT scanners whilst providing better quality images have the disadvantage of not being able to scan the whole anterior segment in one scan.

## Conclusion

In summary we have successfully validated the repeatability of the ZAP software measurements of ASOCT images from patients who have undergone DSAEK surgery. We have also shown that the graft arc length parameters calculated from the software correlate well with the intra operative graft trephine diameter. There will be much value in analyzing the correlation between graft parameters measured on ASOCT and DSAEK outcomes, which may enable us to optimize graft trephine diameter chosen for DSAEK surgery. The use of high resolution ASOCT scans in the future will also provide greater detail with more quantitative measurements that may be useful in studying outcomes and optimizing surgical decisions. The ability to accurately select the ideal donor diameter, to maximize endothelial cell transfer while reducing the risks of anterior chamber crowding and peripheral anterior synechiae, and predict the final refractive outcome, could help to improve graft survival, and visual outcomes, as well as reduce complications in DSAEK surgery.

## Competing interests

The authors declare they have no competing interests.

## Authors’ contributions

GT participated in the design of the study, performed the measurements on the ASOCT images, performed the statistical analysis and drafted the manuscript. MH advised on the design of the study, developed the software used for image measurements and advised on drafting the manuscript. DT performed the surgery on all patients in the study, advised on the design of the study and advised on the drafting of the manuscript. JM conceived of the study and participated in the design and coordination of the study as well as the drafting of the manuscript. All authors read and approved the final version of the manuscript.

## Financial disclosure

National Research Foundation-Funded Translational & Clinical Research (TCR) Program Grant [NMRC/TCR/002 - SERI/2008 - TCR 621/41/2008].

## Pre-publication history

The pre-publication history for this paper can be accessed here:

http://www.biomedcentral.com/1471-2342/12/19/prepub
